# Thomas H. Benton

**Published:** 1857-06

**Authors:** 


### THOMAS H. BENTON.

To be old and to be well, to pass days, and weeks, and months without an ache, or ail, or pain, or grunt, or groan, to eat heartily every meal, to sleep soundly every night, and to enter upon every undertaking with the energy, and determination, and zest of youth, at the age of seventy-five, is a blessing beyond computation; it is a happiness worth more than millions of treasure. In fact, there is many a rich man who would cheerfully part with his wealth if he could purchase back the health of his youth. We knew a man in our childhood, who was familiarly called "Jake Allenthorpe;" he had risen to wealth by long years of laborious attention to business, but following the custom of the times, he was a daily liquor drinker, and as a consequence by no means unusual, he lost his health, and died long before reaching a good old age. On one occasion, when under acute suffering, he said: "I would cheerfully give all I possess, if I could have the health I had when I was a young man and worked for fifty cents a day." There are thousands in this land who could make a like declaration. But it is recorded of Mr. Benton, that, "After a long and continuous tour through New England he returned to Washington on Saturday to leave in a few days for Vermont and elsewhere. (The thermometer having been below zero.) He seems invigorated by the contest with storms, snow sieges, ice barricades, and rather rejoices in the hope that the stuff in him may be put to new trial by fresh exposures. At midsummer he was in the West, traversing Missouri night and day, stumping after the most approved fashion; and now, in midwinter, with the same energy, he is defying the elements under the eternal snows which look down from the White Mountains in the East."

What made the immense difference between William Allenthorpe, the Kentucky farmer, and Thomas H. Benton, the Missouri politician—the difference of uninterrupted good health, and twenty-five years of life besides? The farmer took his glass of Bourbon whiskey every day, the old Roman never took a drop, but from principle lived a life of systematic temperance in all things, except when he made faces at General Kearney at the trial of Col. Fremont.

To live long, and well, and usefully, then, be temperate in all things, remembering that the only certain and effectual way of being temperate in reference to liquor is, NEVER TASTE A DROP.

---

COFFEE.—Just two hundred years ago (1657), a man was prosecuted in London for selling coffee, as a "nuisance and a prejudice in the neighborhood." There are a good many people now, who consider coffee a "nuisance and a prejudice." Are we advancing towards truth, or going back to it?

DOCTORS of almost every school are indebted to the inventive genius of MINTHORN, of this city. His portable gum elastic *syringe*, weighing half a dozen ounces, easily carried in a small pocket, is the most perfectly simple, ingenious, and handy contrivance of the kind we have ever seen; opening and shutting one hand operates it with the utmost ease. Minthorn is, however, greatly chapfallen. While medicated inhalation was all the rage, his versatility immediately fabricated the most convenient apparatus, and he found a ready and large sale for great numbers of them, to the admirers of plausible novelties and resurrected absurdities. In the winter of 1855–6, he sold privately, to persons in New-York, eleven hundred inhalers. During the past winter of 1856 and 1857, he sold but three. He says the word "*inhaler*" is a hissing and a stench wherever he goes.

"*The Scalpel*" is no doubt heartily welcomed back by its old readers to its comely quarterly octavo form. Tart, striking, vigorous, its mirth-provoking and highly instructive pages will make it a sought-for publication. It has one defect, and that so serious an one, that there are thousands of parents who would as soon see on the side-table a volume of Byron, Shelley, Tom Paine, or George Sands, as a number of the *Scalpel*, by reason of its repeated profanities, and ceaseless gibes and ill-natured sarcasms against professors of religion, and ministers especially. If its really talented editor will leave out this obnoxious element in the future "making up" of his wide-famed journal, we believe he would largely add to the number and respectability of his subscribers. If the editor has been more largely injured in any way by the professors or ministers of religion, let him direct his shafts to them individually, and not impugn the motives, sentiments, and conduct of one half of Christendom, for the malice he may owe to a score of derelicts.

Rees' Medical Gazette.—Will the learned editor of this widely-known monthly, by the large circulation of any practical article which he may think proper to write, such as those on *Tobacco* and the *Treatment of Burns*, be stimulated to a more frequent delving into the most capacious pockets of his memory and intelligence for articles alike true, useful, and practical?

